# Dementia and Heart Failure Classification Using Optimized Weighted Objective Distance and Blood Biomarker-Based Features

**DOI:** 10.3390/bioengineering12090980

**Published:** 2025-09-15

**Authors:** Veerasak Noonpan, Supansa Chaising, Georgi Hristov, Punnarumol Temdee

**Affiliations:** 1 Computer and Communication Engineering for Capacity Building Research Center, School of Applied Digital Technology, Mae Fah Luang University, Chiang Rai 57100, Thailand; 6471501501@lamduan.mfu.ac.th; 2 School of Management, Mae Fah Luang University, Chiang Rai 57100, Thailand; supansa.cha@mfu.ac.th; 3 Telecommunications Department, University of Ruse, 7017 Ruse, Bulgaria; ghristov@uni-ruse.bg

**Keywords:** dementia, heart failure, risk factors, blood biomarkers, objective distance, weighting features

## Abstract

Dementia and heart failure are growing global health issues, exacerbated by aging populations and disparities in care access. Diagnosing these conditions often requires advanced equipment or tests with limited availability. A reliable tool distinguishing between the two conditions is essential, enabling more accurate diagnoses and reducing misclassifications and inappropriate referrals. This study proposes a novel measurement, the optimized weighted objective distance (OWOD), a modified version of the weighted objective distance, for the classification of dementia and heart failure. The OWOD is designed to enhance model generalization through a data-driven approach. By enhancing objective class generalization, applying multi-feature distance normalization, and identifying the most significant features for classification—together with newly integrated blood biomarker features—the OWOD could strengthen the classification of dementia and heart failure. A combination of risk factors and proposed blood biomarkers (derived from 10,000 electronic health records at Chiang Rai Prachanukroh Hospital, Chiang Rai, Thailand), comprising 20 features, demonstrated the best OWOD classification performance. For model evaluation, the proposed OWOD-based classification method attained an accuracy of 95.45%, a precision of 96.14%, a recall of 94.70%, an F1-score of 95.42%, and an area under the receiver operating characteristic curve of 97.10%, surpassing the results obtained using other machine learning-based classification models (gradient boosting, decision tree, neural network, and support vector machine).

## 1. Introduction

The proportion of older adults is increasing in every country, possibly due to improvements in education, nutrition, and healthcare. As a result, a new life-course model has been introduced, encompassing early life (younger than 45 years), midlife (45–65 years), and later life (older than 65 years) [1]. Dementia and heart failure are two of the most diagnosed chronic conditions in older adults, frequently co-occurring due to shared risk factors and interconnected biological mechanisms [2,3,4]. With increasing age, individuals are more likely to develop conditions such as high blood pressure, diabetes, high cholesterol, and obesity, all of which are known to increase the risk of heart disease and neurodegenerative diseases [5]. There exists a clear physiological link between these two disorders. For example, heart failure can cause reduced blood flow to the brain, which may accelerate cognitive deterioration. Dementia is also associated with systemic inflammation and metabolic disturbances, which adversely affect cardiovascular function [4]. This two-way interaction highlights the importance of maintaining both cognitive and cardiovascular health in older adults. By managing these comorbidities simultaneously, more effective diagnoses can be developed, allowing for improved treatment outcomes and a higher quality of life for older adults.

Dementia is a growing public concern, impacting the social and economic sectors in terms of medical and social care costs. The global societal cost of dementia was estimated at USD 1.3 trillion in 2019, with costs expected to surpass USD 2.8 trillion by 2030. Additionally, in 2019, the impact on families and caregivers was found to be significant, with an average of 5 h per day spent caring for individuals with dementia. Women were especially impacted; the majority of dementia patients who passed away were female, accounting for up to 65% of cases. Currently, there is no cure for dementia; the primary goal of care is early diagnosis, which is key to optimal management [6]. The increase in the older adult population has given rise to a greater number of dementia prevalence studies [7] that utilize digital healthcare with appropriate technology [8,9,10] and various features [11].

Heart failure is defined as a condition in which the heart has a reduced ability to pump or fill with blood or exhibits structural abnormalities that result in inadequate cardiac output. Heart failure has been described as a global pandemic, affecting approximately 64.3 million people worldwide as of 2017. The prevalence is expected to rise, primarily because individuals live longer after diagnosis due to lifesaving interventions and general improvements in life expectancy. However, the financial burden is significant—in the U.S. alone, the cost of heart failure was estimated at USD 30.7 billion in 2012, and projections suggest that costs could rise by 127% to USD 69.8 billion by 2030 [12]. Given the high prevalence and severe health consequences of heart failure, numerous studies have focused on identifying its risk factors and developing effective prevention strategies.

Biomarkers are generally used for dementia detection. These include cerebrospinal fluid biomarkers, blood-based biomarkers, neuroimaging biomarkers, and genetic biomarkers. Given the high cost of positron emission tomography/magnetic resonance imaging (PET/MRI), blood-based biomarkers offer a practical alternative for detecting dementia. They are also noninvasive and widely accessible, making them suitable for large-scale and repeated assessments. Dementia and heart failure share several risk factors, including body weight, blood cholesterol levels, hypertension, serum lipids, and diabetes [5,6]. Leveraging noninvasive and affordable methods enhances accessibility and equity, particularly in resource-limited settings. However, using such biomarkers from electronic health records (EHRs) remains challenging and requires comprehensive data integration and validation.

Digital healthcare technology is being rapidly implemented among older adults, as it improves monitoring, communication, and health data collection. Currently, healthcare knowledge-based systems, which support personalized care for older adults and their healthcare providers, are widely accessible through smart devices, such as smartphones and smart watches. In addition, innovations in healthcare and medical support are required to enhance personalized healthcare and increase life expectancy. Machine learning (ML) methods have emerged as powerful tools in medical research. In dementia studies, ML methods have been applied in both preclinical and clinical investigations. Examples include studies using various cognitive tests for dementia [13], a deep learning framework to identify dementia from DNA datasets [14], gradient boosting (GB) for dementia classification based on human genetics [15], analysis of the dementia-related gene APOE4 [16], ensemble classifiers for multi-class classification using the Alzheimer’s Disease Neuroimaging Initiative (ADNI) dataset [17], and dementia classification based on PET/MRI [18,19,20], with most datasets derived from the ADNI. ML methods have also been used in the classification of heart failure, such as support vector machines (SVMs) [21,22,23], decision trees (DTs) [24], GB [25], extreme gradient boosting (XGB) [26], and deep learning, across datasets of varying types and sizes. However, ML approaches often require large datasets to perform effectively, and performance can degrade when data are limited. Furthermore, ML methods may struggle with interpretability, which is a key consideration in medical diagnostics. To address these limitations, it is essential to explore an alternative approach that could enhance classification performance while maintaining interpretability.

Distance measurement methods, such as the weighted objective distance (WOD), have been successfully applied in the classification of various diseases, including hypertension [27] and type 2 diabetes [28], which are risk factors for dementia and heart failure [1,29,30,31]. However, the WOD is limited by its reliance on predefined thresholds and fixed values tied to specific diseases, leading to model generalization issues. To address these limitations, this study introduces the optimized weighted objective distance (OWOD). The OWOD enhances the original WOD approach by incorporating an objective function that derives data representations directly from the dataset. It identifies the target values of both dementia and heart failure risk factors and calculates the WOD accordingly. Thus, this study proposes a binary method based on distance measurement for the classification of dementia and heart failure using blood biomarkers and established risk factors from EHRs. The OWOD is expected to strengthen the binary classification of dementia and heart failure by enhancing objective class generalization, applying multi-feature distance normalization, and identifying the most significant features for classification.

## 2. Literature Review

Numerous studies have investigated various aspects of dementia and heart failure. These studies can be categorized into three groups, which are discussed in this section.

### 2.1. Feature Studies of Dementia and Heart Failure

Several studies have focused on the risk factors for both dementia and heart failure. Dementia risk factor studies can be divided into three groups. The first group encompasses the relationship between dementia and body weight [32], blood cholesterol [33], and hypertension [29,30,31]. Such studies have demonstrated significant associations between dementia development and various clinical parameters that can be obtained from routine physical check-ups and blood tests. These include hypertension [29,30,31], body weight [32], blood cholesterol levels [33], serum lipids [34], and diabetes [1]. The second group comprises preclinical studies, such as speech assessment [35,36], lifestyle activity [37], handwriting assessment [38,39], sleep disturbances [40,41], sex differences [42], physical assessment, risk score models [43,44,45], and hearing loss [1]. The third group includes brain imaging clinical studies using PET/MRI [19].

Similarly, heart failure studies can be categorized into three groups. The first group identifies risk factors such as age [46,47], sex [46,47], hypertension [46], hypercholesterolemia [47], low-density lipoprotein cholesterol (LDL-C) [46], diabetes mellitus [46], overweight/obese [47], elevated body mass index (BMI) [46], smoking [46], low physical activity [46], family history of premature cardiovascular disease (CVD) [47], chronic kidney disease [47], and lipid biomarkers [47]. Key risk factors for heart failure—such as obesity, hypercholesterolemia, hypertension, lipid biomarkers, diabetes, sex, and age—are also strongly associated with dementia [12,29,30,31,46,47]. The second group includes studies on preclinical heart failure, such as the incidence of preclinical heart failure in healthy community individuals using health status records during visits. These include age, BMI, systolic blood pressure (SBP), diastolic blood pressure (DBP), heart rate, diabetes, hypertension, obesity, hyperlipidemia, and echocardiogram [48]. Some studies assess the risk of heart failure based on preclinical information [47]. The third group comprises studies on the clinical identification and prevention of heart failure, such as heart disease identification using ML methods [21,22,23], such as DT [24], GB [25], and XGB [26].

While the reviewed studies provide valuable insights across the dementia and heart failure risk continuum—from risk factor identification to preclinical and clinical assessments—several limitations and potential biases warrant consideration. Risk factor studies often rely on observational or retrospective data, which may be subject to confounding variables and lack causal inference. Preclinical studies may introduce cultural or educational biases, limiting generalizability across diverse populations. Furthermore, risk score models in the case of dementia, although practical, can oversimplify complex disease pathways and may not fully capture interactions between multiple features. Clinical studies using advanced equipment and laboratory tests are typically conducted in well-resourced settings, potentially excluding underrepresented populations and limiting real-world applicability. These limitations underscore the need for integrative, accessible, and representative approaches in the classification of dementia and heart failure, balancing precision with inclusivity.

Although dementia and heart failure often co-occur and share multiple risk factors, their distinct clinical manifestations and biomarker profiles justify the use of a binary classification model. Developing such a model is challenging, as it must differentiate between two interrelated conditions with overlapping etiologies yet diverging diagnostic and therapeutic pathways—particularly in cases where early symptoms are ambiguous. The dementia and heart failure risk factors identified in previous studies are shown in Table 1.

This study proposes a new feature and method for classifying dementia and heart failure, with blood biomarkers mainly used to construct the binary classification model. Two groups of blood-oriented features were used in this study. The first group consisted of features associated with dementia and heart failure development from existing works, termed related risk factors (R). The second group consisted of blood biomarker features also obtainable from blood testing, but with no established associations with the two conditions; these were newly proposed for this study and termed potential features (P). Therefore, the features used to develop the classification model were derived from both existing risk factors and a new set of blood biomarker-oriented features. The integration of the new set of features was expected to sharpen the boundary of classes within the dataset.

### 2.2. ML-Based Classifications for Dementia and Heart Failure

Several ML models have been proposed for constructing classification models from different types of data. For dementia studies, the models can be categorized into two groups. First are the models that use imaging datasets for classification [18,19,20,49,50,51,52]. The images are usually obtained from advanced laboratories and tend to have high resolutions, necessitating sophisticated models, such as deep learning-based models. The limitations of imaging-based studies include the need for advanced instrumentation and a sufficiently large sample size to ensure reliable results—factors that are often difficult to achieve in practical healthcare settings. Second are the models that use nonimaging datasets for classification [21,22,23,27,28,43,53]. Such studies utilize a range of biomarkers, such as demographic and psychiatric information [43], DNA methylation profiles [14], genetic data from ADNI-1 [15], and medical attributes [27,28,54].

For heart failure studies, SVMs have been widely adopted across different datasets, achieving prediction accuracies between 92.37% and 98.47% depending on preprocessing techniques, such as principal component analysis or hybrid feature selection [21,22,23]. DT, while simpler, has still proven effective, with an accuracy of 93.00% on Kaggle heart failure data [24]. More recent approaches have leveraged physiological signals—such as electrocardiograms—processed through recurrent neural networks and deep learning models—producing high accuracies of 99.86% [53] and 97.93% [51], respectively. These results revealed the effectiveness of the incorporation of time-series and image-based representations of cardiac signals, which appear to significantly enhance the accuracy and robustness of heart failure classification models.

Despite the high comorbidity rate of heart failure and dementia, few studies have addressed their joint classification. Previous work by the team of the corresponding author explored both binary and multi-class classification models. For binary classification, an extra trees model with data balancing achieved 89.11% accuracy on a small dataset (4297 records) [55], increasing to 96.47% on a larger dataset (14,763 records) using a data enrichment framework [54]. For multi-class classification, GB with 59 features across more than 16,000 records classified heart failure, aortic stenosis, and dementia with 83.81% accuracy [27]. XGB with 108 augmented features on 26,474 records achieved 91.42% accuracy [26]. These studies highlight the importance of large-scale, feature-rich data and robust ML algorithms for classification problems. A persistent challenge in applying ML to medical/healthcare data is the insufficiency of clinical data, as many datasets are incomplete or imbalanced, limiting the model’s ability to generalize and increasing the risk of overfitting, particularly in complex classification tasks. In addition, the complexity and limited interpretability of such models necessitate the development of more transparent methods, such as distance measurements.

### 2.3. Classification with Distance Measurements

Classification using distance measurements is expected to outperform ML-based models in these specific scenarios as it relies on direct comparisons between feature vectors, making it more robust, less sensitive to overfitting, and more interpretable. This is especially the case when the feature space is well defined and clinically meaningful. Classification methods using distance measurements have been introduced and widely applied in various domains over the decades. These include Euclidean distance [56], Manhattan distance [57], distance measures on fuzzy c-means algorithms [58], and objective distance [59,60,61]. Recently, a WOD [27] was proposed to solve personalized care for older adults with hypertension. This method measures the distance between the current health status of an individual and their defined level of hypertension and then generates personalized feedback based on the distances obtained. Additionally, the average weighted objective distance (AWOD) [28] has been applied for predicting type 2 diabetes. Both the WOD and AWOD leverage the concepts of objective distance, demonstrating their potential for measuring the distance between an individual’s status and expected health goals. The WOD utilizes weighting factors derived from information gain to prioritize these factors in distance calculations. The AWOD builds upon this framework by focusing on the average distances between features and is particularly effective for binary classification problems. However, both methods have limitations: the WOD often struggles with comparability in distances due to its reliance on static weighting, while the AWOD, despite improving upon the WOD, may overlook the complexities in the interactions between risk factors. These limitations highlight a critical gap in the effective modeling of the influence of diverse health conditions. To address these challenges, this study proposes the OWOD, which modifies the distance metric for each feature, enhances comparability, and employs a refined objective function derived directly from the dataset of dementia and heart failure. This new distance aims to improve the performance of the classification model and promote model generalization.

### 2.4. The Proposed Study

The proposed OWOD introduces an optimized target value, defined as the point of maximum divergence between class histograms. This value represents the most discriminative threshold reflecting the class boundaries and is used to determine the optimal decision point for classification. Compared with the WOD and AWOD, the OWOD enhances the modification of each feature’s distance values to facilitate comparability. It also improves on the previous distances’ predefined and standard values, in terms of finding the objective function that represents values from the dataset, thereby enhancing generalization.

Similar to the WOD and AWOD, the weighting factors of the OWOD are derived from information gain. Information gain, derived from entropy, measures uncertainty or impurity in a dataset. Entropy quantifies the level of uncertainty or disorder within data. In the classification problem, entropy is primarily utilized to determine the irrelevant attributes of a dataset [62]; accordingly, information gain is one of the most popular methods for feature selection problems [63,64]. Information gain is used as a novel feature selection for text classification problems [65]. It reduces the dimensionality of features available in the document for improving classification performance. In addition, it is applied as a weight coefficient to enhance the effectiveness of the classification algorithm [66]. Weighting attributes can improve classification accuracy [67] by prioritizing attributes from the least to the most important.

For the current study, the classification model using the OWOD was constructed with an equal number of dementia and heart failure records, ensuring both conditions were treated as equally important. This approach addressed the limitations of previous measurements, including the WOD and AWOD, by refining distance calculations and employing a more effective objective function derived from the dataset. The risk factors and additional blood biomarker features were employed to sharpen the boundary between the two dataset classes: patients with dementia and patients with heart failure. It is hypothesized that new blood biomarker features and the OWOD can promote generalization and provide better classification performance compared with other ML-based binary classification models.

## 3. Research Methodology

**Figure 1:**
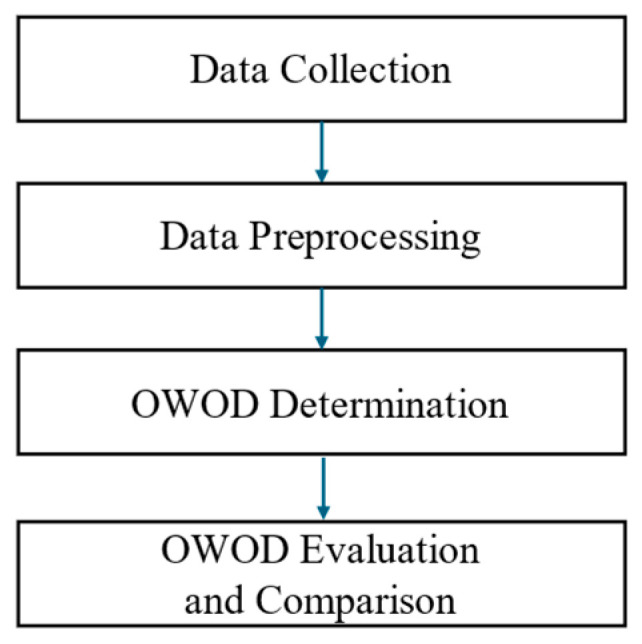
Research methodology consisting of four main processes: data collection, data preprocessing, optimized weighted objective distance (OWOD) determination, and OWOD evaluation and comparison.

This study consisted of four main procedures, as shown in Figure 1.

The details of each procedure are discussed in the following sections.

### 3.1. Data Collection

The dataset used in this study is the EHR from the Chiang Rai Prachanukroh Hospital, Chiang Rai, Thailand, spanning the years 2016 to 2022. The dataset includes blood tests and clinical records of individuals aged 60 and over, comprising a total of 12,222 records (dementia = 5563; heart failure = 6659). No individual was diagnosed with both diseases simultaneously. Table 2 shows the features used.

As previously discussed (and shown in Table 2), the features used in this study were divided into two groups: R and P. The R group (including body weight, blood cholesterol, and blood pressure) was employed as it was associated with dementia and heart failure in the existing research. The P group was obtained through blood tests. Physical activity was not considered a risk factor in this study due to insufficient data; this includes smoking and alcohol history.

### 3.2. Data Preprocessing

**Figure 2:**
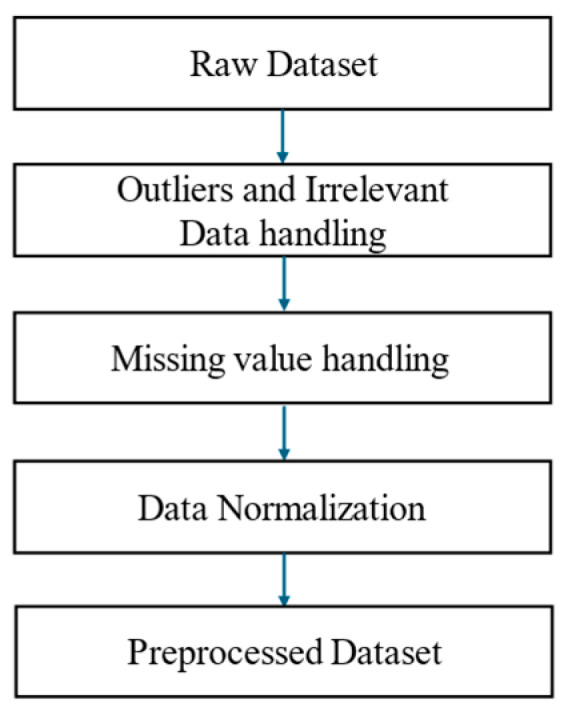
Data preprocessing workflow comprising outlier and irrelevant data handling, missing value imputation, and data normalization.

The dataset originally consisted of 12,222 records, including 5563 dementia records and 6659 heart failure records. The raw dataset subsequently went through the preprocessing procedure shown in Figure 2. Following this, the final dataset was balanced to include 10,000 records, with 5000 dementia and 5000 heart failure cases.

Prior to normalization, erroneous or invalid entries were addressed by handling outliers and irrelevant data. This included filtering or correcting values deviating significantly from the expected distribution or deemed implausible due to data entry errors (e.g., extremely low or high body weights). Missing values were addressed using mode imputation, based on the most frequent values observed in the data distribution. Data normalization was applied as part of the preprocessing workflow to ensure consistency and reliability before proceeding to OWOD determination. This step involved adjusting the raw data to a standard scale, typically by standardizing values with a mean of 0 and a standard deviation of 1, or by scaling them to a defined range. Such preprocessing ensured that the final dataset was statistically valid and suitable for subsequent analysis.

**Figure 3:**
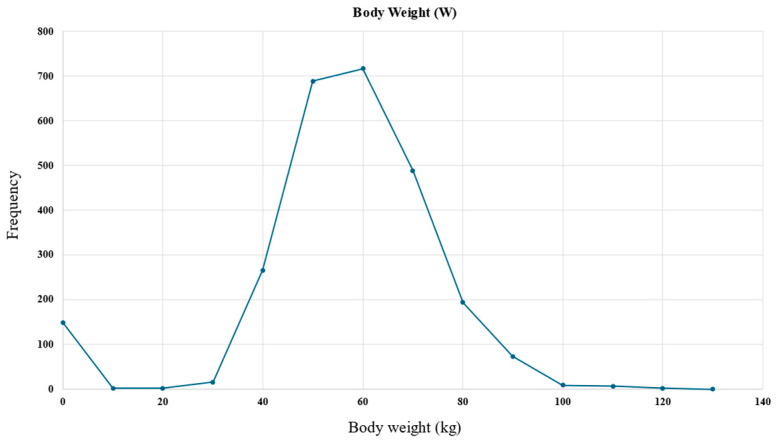
Body weight feature histogram used in the data preprocessing step to handle outliers and missing values.

An example histogram of the body weight feature is presented in Figure 3, demonstrating its applicability in the data preprocessing step.

It can be observed in Figure 3 that weight values within the 0–40 kg range were erroneous entries. To address this, the data were filtered to exclude values falling outside the expected distribution range. The missing body weight entries were attributed to the most commonly occurring value, which was 60 kg.

### 3.3. The OWOD Concept

**Figure 4:**
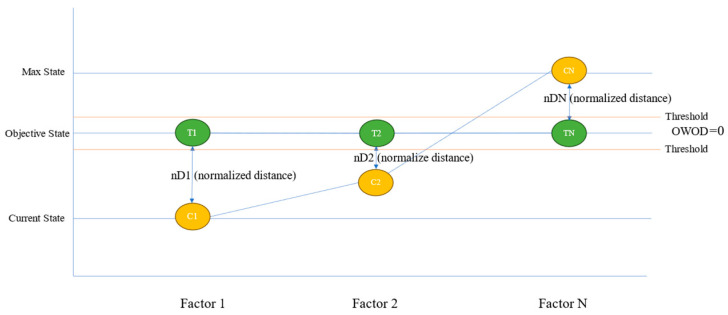
The concept of the optimized weighted objective distance (OWOD), a weighted distance-based measure between current and objective states, reflecting the influence of each feature at the individual level.

The underlying principle of the OWOD method is based on the distance between current and objective states, with the weights derived from information gain. The weights reflect the actual impact on models and represent the diverse health conditions of individuals, as well as the general diagnostic procedures used by healthcare professionals. This concept is illustrated in Figure 4.

Figure 4 shows that three main steps are required for OWOD determination. The first step involves determining the objective value for distance calculation. The objective value ($T_{i}$) is the state level of each feature (i), which represents the group of dementia patients in the dataset. The current value ($C_{i}$) is the current state for each feature. The acceptable value ($A_{i}$) is the health status level for each feature acceptable for an individual. All three level values are used to determine the OWOD.

### 3.4. OWOD Determination

**Figure 5:**
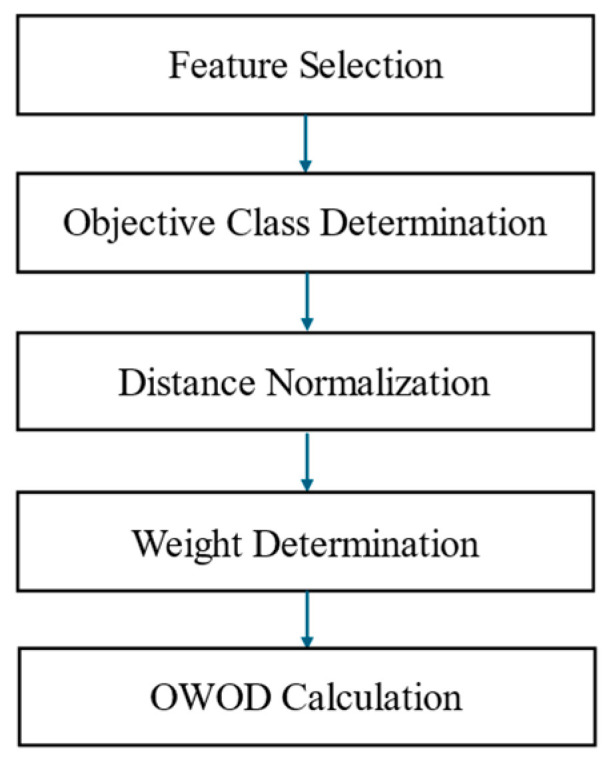
The optimized weighted objective distance (OWOD) determination process comprising feature selection, objective class determination, distance normalization, weight determination, and OWOD calculation.

The OWOD determination process is illustrated in Figure 5.

The OWOD algorithm consists of five processes, beginning with feature selection, where the number of features used in the calculation is determined. The objective value represents the target level of each feature derived from the dataset. The next step involves calculating the normalized distance between the current state and the objective state. Entropy and information gain are then used to compute the weight for each feature. Factor weighting is determined based on information gain, and the final step involves calculating the OWOD.

#### 3.4.1. Feature Selection

This study utilized a mixed selection of R and P features, testing various sets of 8, 12, 16, and 20 features to determine the feature combination of the best model. These selections were intended to investigate how varying levels of feature dimensionality influence OWOD model performance. These numbers represented incremental increases that helped examine the trade-off between feature richness and overall classification performance.

#### 3.4.2. Objective Class Determination

**Figure 6:**
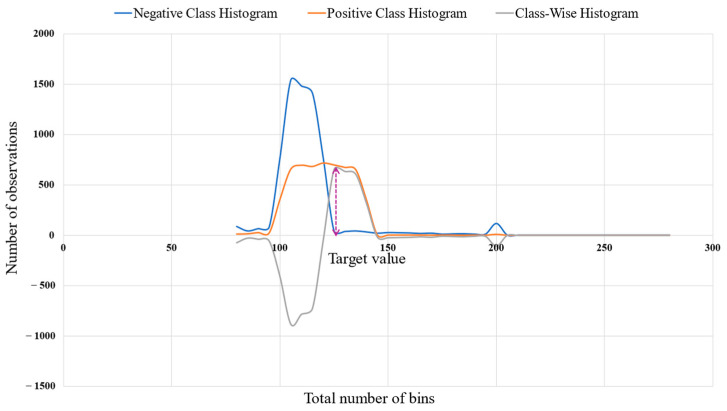
Determination of the target value (dotted arrow) for each feature based on class-wise histogram distributions, illustrating the distributional differences between positive and negative classes.

To calculate the OWOD, the objective class was determined. A histogram was used to find the data distribution of the objective class. The histogram was calculated using Equation (1).

(1) $$n=\sum_{i=1}^{k}m_{i}\;$$

where *n* refers to the total number of observations, *k* refers to the total number of bins, and $m_{i}$ refers to the histogram data. The target value was defined as the value of observation *k* corresponding to the maximum positive difference between the histograms of the positive and negative classes. This objective value was determined from the class-wise histogram distribution presented in Figure 6.

The target value of this feature was calculated using Equation (2).

(2) $$T_{mi}=\mathrm{Mode}\;\left(\mathrm{Max}\;\left(\;\mathrm{Histogram}\;\left(\mathrm{PositiveClass}\right)-\mathrm{Histogram}\;\left(\mathrm{NegativeClass}\right)\right)\right)$$

where $T_{mi}$ denotes the target value of the observation feature and i corresponds to the point of maximum difference between the positive and negative class distributions.

#### 3.4.3. Distance Normalization

The distance calculation method was used to determine the distance between the current and objective state features. The distance was calculated using Equation (3).

(3) $$\mathrm{dTC}=\sqrt{{\left(T-C\right)}^{2}\;}$$

where T refers to the target value of attributes, C refers to the current state value of attributes, and dTC refers to the distance between the target and current states.

(4) $$\mathrm{dTA}=\sqrt{{\left(T-A\right)}^{2}\;}$$

where T refers to the target value of attributes, A refers to the acceptable state value of attributes, and dTA refers to the distance between the target and acceptable states.

Data from multiple features were combined in the calculation. As distance was a key measurement, any bias could lead to issues in the final OWOD calculation. The normalization method was therefore applied. Normalization was calculated using Equation (5).

(5) $$\mathrm{ndTC}=\;\frac{dTC}{A_{max}}$$

where dTC is the distance from Equation (3), $A_{\max}$ is the acceptable value and the maximum value, and ndTC is the normalized distance between the target and current states. This is shown in Equation (6).

(6) $$\mathrm{ndTA}=\;\frac{dTA}{A_{max}}$$

where dTA is the distance from Equation (4), $A_{\max}$ is the acceptable value and the maximum value, and ndTA is the normalized distance between the target and acceptable states.

The normalized distance ratio used for the weight calculation rTC is the normalized distance ratio of the normalized distance between the target and current states compared to the total normalized distance. This is shown in Equation (7).

(7) $$\mathrm{rTC}=\;\frac{ndTC}{ndTA+ndTC}$$

rTA is the normalized distance ratio of the normalized distance between the target and acceptable states compared to the total normalized distance. This is shown in Equation (8).

(8) $$\mathrm{rTA}=\;\frac{ndTA}{ndTA+ndTC}\;$$

#### 3.4.4. Weight Determination

To determine the entropy of the target class with respect to all attributes, an equal proportion of the target class was initially determined using Equation (9). This resulted in an equal proportion of positive and negative classes.

(9) $${Oc}_{+}={Nc}_{-}=\frac{Na}{N}\;$$

where ${Oc}_{+}$ is the value of the equal proportion for the positive class (+) with respect to all attributes, ${Nc}_{-}$ is the value of the equal proportion for the negative class (−) with respect to all attributes, Na is the total number of attributes, and N is the total number of target classes.

Next, the fraction of the target class was determined using Equation (10). This value represents the fraction of the chance of being in a positive or negative class with respect to all attributes.

(10) $${\;fOc}_{+}=\frac{{Pe}_{+}}{Na}={fNc}_{-}=\frac{{Pe}_{-}}{Na}\;$$

where $fOc_{+}$ represents the fraction of the positive class (+) and $fNc_{-}$ represents the fraction of the negative class (−), in which both classes are equal.

The weight determination relies on entropy and information gain.

- Entropy.

Shannon Entropy, an information theory method, quantifies the average level of information, surprise, uncertainty, or complexity for a given random variable based on its historical occurrences. Entropy ($EP\left(X\right)$) was calculated using Equation (11).

(11) $$EP\left(X\right)=-\sum_{i=1}^{n}P{(x}_{i})\;\log_{2}P\left(x_{i}\right)\;$$

where X is the class of attributes, $P{(x}_{i})$ is the proportion of the samples belonging to class X, and $\log_{2}$ is log based 2.

- Information Gain.

Information gain is the reduction in entropy produced by partitioning a set with attributes a and finding the optimal candidate that produces the highest value. The information gain ($IG\;\left(T,a\right)$) was calculated using Equation (12).

(12) $$IG\;\left(T,a\right)=H\left(T\right)-H\left(T|a\right)$$

where T is a random variable and H(T|a) is the entropy of T given the value of an attribute a.

The split information value is a positive integer that describes the potential worth of splitting a branch from a node. This, in turn, is the intrinsic value the random variable possesses and is used to remove bias from the information gain ratio calculation. The split information value ($S\left(X\right)$) was calculated using Equation (13).

(13) $$S\left(X\right)=-\sum_{i=1}^{n}\;\frac{N(t_{i})}{N(t)}*\;\log_{2}\;\frac{N\left(t_{i}\right)}{N\left(t\right)}\;$$

where X is a discrete random variable with possible value $X_{i}$ and $N(t_{i})$ is the number of times that $t_{i}$ occurs, divided by the total event count $N\left(t\right),$ where t is the set of events.

The information gain ratio (IGR (T,a)), the ratio of the information gain and the split information value for the T variable $S\left(T\right).\;IGR\;(T,a)$, was calculated using Equation (14).

(14) $$IGR\;\left(T,a\right)=\frac{IG(T,a)}{S(T)}\;$$

The weight information ($IW_{i}$) was calculated using Equation (15).

(15) $$IW_{i}=\frac{R_{eg}}{\sum_{i=1}^{N}R_{eg}}\;$$

where $R_{eg}$ is the ratio of entropy from Equation (11) and the information gain from Equation (14), and $W_{i}$ is the calculated weight information.

#### 3.4.5. OWOD Calculation

To determine the OWOD of all attributes, the WOD of each attribute $\left({owoD}_{i}\right)$ was first determined using Equation (16). Let $T_{mi}\;=\;t_{mi},\;A_{i}\;=\;a_{i},\;C_{i}\;=\;c_{i}$.

(16) $${owoD}_{i}=W_{i}*n\left|\sqrt{{{(T}_{mi}-A_{i})}^{2}}-\sqrt{{{(T}_{mi}-C_{i})}^{2}\;}\right|\;$$

After obtaining the ${owoD}_{i}$ of each person, the normalized WOD of each attribute (${nowoD}_{i}$) was determined using Equation (17).

(17) $${nowoD}_{i}=\frac{{owoD}_{i}-{owoD}_{min}}{{owoD}_{max}-{owoD}_{min}}\;$$

where ${owoD}_{\max}$ is the maximum OWOD among all attributes and ${owoD}_{\min}$ is the minimum OWOD among all attributes.

Therefore, the OWOD of all attributes for each person (${OWOD}_{i}$) was determined using Equation (18).

(18) $${OWOD}_{i}=\frac{\sum_{i=1}^{\mathrm{Na}}{nowoD}_{i}\;}{Na}$$

where ${OWOD}_{i}$ denotes the OWOD of all attributes for the *i*th individual.

To identify the class based on the obtained ${OWOD}_{i}$, the following condition was applied, as shown in Equation (19):

(19) $${OWOD}_{i}\;=\;\left\{\;\begin{array}{c} \mathrm{Negative}\;\mathrm{class},\;\mathrm{if}\;{OWOD}_{i}>\;{OWOD}_{c}\;\\ \mathrm{Positive}\;\mathrm{class},\;\mathrm{if}\;{OWOD}_{i}\;\leq \;{OWOD}_{c}\; \end{array}\right.$$

where ${OWOD}_{c}$ is the cut-off OWOD calculated from threshold ${Ts}_{mi}$ of all features. ${Ts}_{mi}$ refers to the threshold of the target values for each feature and is defined as the deviation of 5% above and below the $T_{mi}$ of all features. For this study, the negative class was heart failure, and the positive class was dementia.

#### 3.4.6. OWOD Algorithm

The algorithm used to perform the OWOD calculation is presented in the pseudocode shown in Algorithm 1 as follows:
**Algorithm 1:** OWOD calculation**Input:** List of selected features (**F**) **Output:** Table of computed OWOD values 1: **Initialize** storage results 2: **For** each sample **S** in the dataset **do** 3: **For** each feature **F do** 4: **Retrieve C** (current level), **T** (target level), **A** (acceptable level) 5: **Compute** Euclidean distances:           dTC ← √((T − C)^2^)           dTA ← √((T − A)^2^) 6: **Compute** Normalized distances:           ndTC ← dTC/A           ndTA ← dTA/A 7: **Compute** Normalized distance ratios:           rTC ← ndTC/(ndTC + ndTA)           rTA ← ndTA/(ndTC + ndTA) 8: **Compute** Entropy, information gain, and gain score:           EP ← **calculate** entropy (rTC, rTA)           IG ← **calculate** information gain (EP)           G ← **calculate** gain (IG) 9: **Compute** Entropy–gain ratio and weight:           rEG ← EP/G           SrEG ← **sum** (rEG)           W ← rEG/SrEG 10: **Compute** Distance difference, weight*distance and normalize:           DF ← |ndTC − ndTA|           WD ← W × DF           MxWD ← **max** (WD), MnWD ← **min** (WD)           nWD ← (WD − MnWD)/(MxWD − MnWD) 11: **Compute** OWOD:           OWOD ← **average** (nWD) 12: **Store** S, F, and OWOD values 13: **End for** 14: **End for** 15: **Return** OWOD result table

#### 3.4.7. Sample Calculation

This section demonstrates a sample calculation of the OWOD for classifying a potential group of people with dementia and a group with heart failure. To compute the OWOD, both the target and acceptable levels for each feature are applied. The target level represents the desired or optimal value for each feature; the acceptable level indicates the range of values considered tolerable. The acceptable level for each feature is defined as the standard deviation from the average value of the feature.

**Figure 7:**
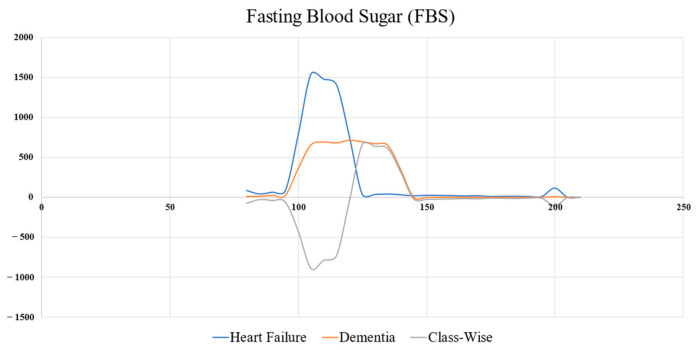
Target value determination of FBS, which is derived from a class-wise histogram distribution between the positive and negative classes.

The objective function relies on obtaining the boundary of two classes by plotting the histogram of each feature, including the histogram of the objective and the other class; the target level is calculated by histogram subtraction of the two classes (grey line in Figure 7). The peak of the grey line indicates the target value.

As illustrated in Figure 7, the target level of the fasting blood sugar (FBS) ($T_{\mathrm{FBS}}$) can be determined using Equation (2), resulting in a value of 130. Accordingly, when $T_{\mathrm{FBS}}$ = 130, the acceptable level ($A_{\mathrm{FBS}}$) = 280. More samples of target and acceptable levels are shown in Table 3. Table 4 shows the samples of records used to calculate the OWOD.

The classification of dementia and heart failure using the proposed measurement can be illustrated by applying the information of Person No. 1. The datasets related to the current health status of Person No. 1 are shown in Table 4. The target and acceptable levels of all features are shown in Table 3. The sample attributes comprise eight features. The target group is an objective class (OC) group and a non-objective (NC) class group. OC refers to the positive class (with dementia) and NC to the negative class (with heart failure). Following Algorithm 1, the sample calculation of the OWOD for identifying Person No. 1 is demonstrated below.

To determine the entropy of the target group with respect to all features, an equal proportion of the target group was initially determined using Equation (9). The value of the OC group $\left({Oc}_{+}\right)$ and NC group $\left({Nc}_{-}\right)$ is equal to 4, as follows:

$${Oc}_{+}=\;{Nc}_{-}=\frac{8}{2}\;=4$$

Next, the fraction of the target group with respect to all features was determined using Equation (10). The fraction of the OC group (${fOc}_{+}$) and the NC group (${fNc}_{-}$) with respect to all features is as follows:

$${fOc}_{+}{=\frac{4}{8}\;fNc}_{-}=\frac{4}{8}$$

Thereby, the entropy of the target group with respect to all features ($EP\left(C\right)$) was determined using Equation (11). The value of $EP\left(C\right)$ is equal to 1 as follows:

$$EP\left(C\right)=\;-\frac{4}{8}\times \log_{2}\left(\frac{4}{8}\right)-\frac{4}{8}\times \log_{2}\left(\frac{4}{8}\right)=1$$

To determine the entropy of each feature, an example of the entropy calculation for the weight factor is presented.

Firstly, the current distance (${dTC}_{w}$) and acceptable distance ${(dTA}_{w})$ were determined using Equations (3) and (4), respectively. According to the condition (*i*),$\;T_{w}=t_{w}=55,\;C_{w}=c_{w}=65$, and $A_{w}\;=a_{w}=90$, the ${dTC}_{w}$ and the ${dTA}_{w}$ are equal to 10 and 35 as follows:

$${dTC}_{w}\;=\sqrt{{\left(55-65\right)}^{2}\;}=10$$

$${\;dTA}_{w}=\sqrt{{\left(55-90\right)}^{2}\;}=35$$

The distances of ${dTC}_{w}$ and ${dTA}_{w}$ must be normalized and converted to percentages using Equations (5) and (6), respectively, due to the different range of each feature.

$${ndTC}_{w}=\left(\frac{10}{90}\right)*100=11.11$$

$${\;ndTA}_{w}=\left(\frac{35}{90}\right)*100=38.89$$

Next, the proportion of the acceptable distance ${(rTA}_{w})$ and the current distance ${(rTC}_{w})$ were calculated using Equations (7) and (8), respectively. The ${rTA}_{w}$ and ${rTC}_{w}$ values are equal to 0.22 and 0.78, as follows:

$${rTC}_{w}\;=\;\frac{11.11}{38.89+11.11}\;=0.22$$

$${rTA}_{w}=\frac{38.89}{38.89+11.11}=0.78$$

The fractions of the OC group ${(fOc}_{W+})$ and the NC group ${(fNc}_{W-})$ are as follows:

$${fOc}_{w+}=\;\frac{0.22}{0.78+0.22}\;=\;\frac{\;0.22}{1}$$

$${fNc}_{w-}=\frac{0.78}{0.78+0.22}=\frac{\;0.78}{1}$$

Thereby, the entropy of W feature $\left(EP\left(C_{w}\right)\right)$ was determined using Equation (11). The $EP\left(C_{w}\right)$ is equal to 0.76, as follows:

(20) $$EP\left(C_{w}\right)=-\;\frac{\;0.22}{1}\;*\;{log}_{2}\left(\frac{\;0.22}{1}\;\right)-\;\frac{\;0.78}{1}*\;{log}_{2}\left(\frac{\;0.78}{1}\right)=0.76$$

Thus, the entropies of SBP, DBP, FBS, TGS, TC, HDL, and LDL are equal to 0.92, 0.86, 0.34, 0.99, 0.87, 0.99, and 1.00, respectively.

To determine the information gain of the target group with respect to all features, the entropy of all features was calculated using Equation (12). The $IG\left(C_{a}\right)$ is equal to 0.84, as follows:

$$\begin{array}{ll} IG\left(C_{a}\right) & =0.76\;*\left(\frac{\;0.22+0.78}{8}\right)\;+0.92\;*\left(\frac{\;0.33+0.67}{8}\right)\\ & +0.86\;*\left(\frac{\;0.29+0.71}{8}\right)+0.34\;*\left(\frac{\;0.06+0.94}{8}\right)\\ & +0.99\;*\left(\frac{\;0.46+0.54}{8}\right)+0.87\;*\left(\frac{\;0.29+0.71}{8}\right)\\ & +0.99\;*\left(\frac{\;0.57+0.43}{8}\right)\;+1.0\;*\left(\frac{\;0.5+0.5}{8}\right)\\ & =0.84 \end{array}$$

Thereby, the information gain of the target group with respect to all features $\left(G\left(C,\;T\right)\right)$ is equal to 0.16 as follows:

$$G\left(C,T\right)=\;1-0.84\;=0.16$$

To determine the weight of each feature, the weight calculation for the weight factor is presented as an example. The significant ratio value of the weight factor ($R_{w}$) was calculated, which is equal to 4.75, as follows:

$$R_{W}=\frac{0.76}{0.16}=4.75$$

Thus, $R_{sbp}=5.80$, $R_{dbp}=5.45$, $R_{\mathrm{FBS}}=2.13$, $R_{tgs}=6.26$, $R_{tc}=5.52$, $R_{hdl}=6.22$, and $R_{ldl}=6.32$.

Thereby, the weight of the weight factor ${(W}_{w})$ was determined using Equation (15), which is equal to 0.11, as follows:

$$W_{w}=\frac{4.75}{4.75+5.80+5.45+2.13+6.26+5.52+6.22+6.32}=0.11$$

Thus, $W_{sbp}=0.14$, $W_{dbp}=0.13$, $W_{\mathrm{FBS}}=0.05$, $W_{tgs}=0.15$, $W_{tc}=0.13$, $W_{hdl}=0.15,\;\mathrm{and}\;W_{ldl}=0.15$.

To determine the OWOD of all features, the WOD of the weight factor (${owoD}_{w}$) was calculated using Equation (16), which is equal to 3.15, as follows:

$${owoD}_{w}=0.11*\left|\;38.89-11.11\right|=3.15$$

Thus, ${owoD}_{sbp}=1.44,{\;owoD}_{dbp}=1.75$, ${owoD}_{\mathrm{FBS}}=2.50,\;{owoD}_{tgs}=0.46$, ${owoD}_{tc}=1.75$, ${owoD}_{hdl}=1.05,$ and ${owoD}_{ldl}$ = 0.

From the values of owoD among the eight features, ${owoD}_{max}$ is 3.15 and ${owoD}_{min}$ is 0. The normalized WOD of weight (${nwD}_{w}$) was determined using Equation (17), which is equal to 1.00, as follows:

$${nowoD}_{w}=\frac{3.15-0}{3.15-0}=1.00$$

Thus, ${nowoD}_{sbp}=0.46,$ ${nowoD}_{dbp}=0.55$, ${nowoD}_{\mathrm{FBS}}=0.79$, ${nowoD}_{tgs}=0.15,$ ${nowoD}_{chol}=0.55,{nowoD}_{hdl}=0.33,$ and ${nowoD}_{ldl}=0$.

Therefore, the OWOD of all features classifying the group of Person No. 1 ($O{WOD}_{1}$) is equal to 0.48, as follows:

$$O{WOD}_{1}=\frac{1.00+0.46+0.55+0.79+0.15+0.55+0.33+\;0}{8}=0.48$$

According to the conditions, ${OWOD}_{1}=0.48;$ before identifying the class of ${OWOD}_{1}$, ${OWOD}_{c}$ was calculated with a similar algorithm by changing the current status value ($C_{i}$) to the threshold value (${Ts}_{mi}$) of all features, as illustrated in this calculation of an eight-feature dataset. The list of ${Ts}_{mi}$ values is as follows: ${Ts}_{mi-w}=52.5$, ${Ts}_{mi-sbp}=142.5,\;{Ts}_{mi-dbp}=80.75$, ${Ts}_{mi-\mathrm{FBS}}=123.5$, $\;{Ts}_{mi-tgs}=133$, ${Ts}_{mi-tc}=190$, ${Ts}_{mi-hdl}=52.25,\;and\;{Ts}_{mi-ldl}=128.25$. After determining that the ${OWOD}_{c}$ value = 0.56, it could be identified that Person No. 1 was in the positive class (0.48 ≤ 0.56) and, therefore, in the dementia group.

#### 3.4.8. OWOD Evaluation and Comparison

**Figure 8:**
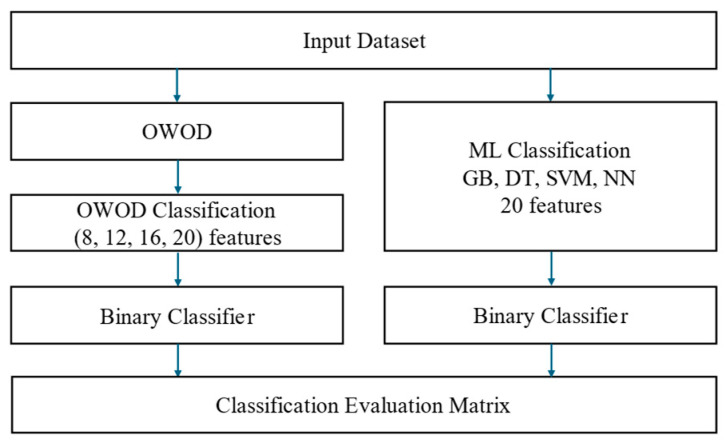
The optimized weighted objective distance (OWOD) evaluation framework for determining the optimal number of features used in constructing the OWOD and comparing classification performance with other machine learning (ML) models.

This section presents the OWOD evaluation process, as illustrated in Figure 8. The OWOD method is compared with the OWOD with differences in feature selection and other ML classification models.

The impact of increasing the number of features (8, 12, 16, 20) was investigated to assess how varying levels of feature dimensionality influence the performance of the OWOD model. The best-performing OWOD model, identified based on the optimal number of features, was compared with several ML models, including GB, DT, neural network (NN), and SVM. For training and testing processes, model performance was assessed using standard evaluation metrics: accuracy, precision, recall, F1-score, and area under the receiver operating characteristic curve (AUC-ROC). A 5-fold cross-validation was applied to the training dataset (80%) to ensure robust validation, and the results were compared with the testing performance on the remaining 20% of the data.

The OWOD algorithm builds upon the previously established WOD method requiring predefined feature ranges and target values, which must be manually specified based on expert knowledge. The OWOD eliminates this requirement by directly deriving the feature ranges and target values from the dataset through an objective function. This data-driven approach makes the OWOD particularly well suited for classifying dementia and heart failure, where biomarkers and their optimal ranges are often poorly established. Consequently, the WOD is not applicable for direct comparison with the OWOD in this context.

## 4. Results and Discussion

This section presents the results of the proposed measurement for classifying groups of patients with dementia and heart failure. The input data used for training and validating consisted of 8000 records containing risk factors and blood biomarker features for both groups. These records were used to calculate the OWOD values and train the ML models. The evaluation was performed using 2000 unseen records to assess the classification performance of both the OWOD approach and the ML models.

### 4.1. Optimal Feature Dimension Results

Table 5 presents the results of the OWOD classification model using varying numbers of selected features, based on 8000 sample records representing 80% of the dataset.

**Figure 9:**
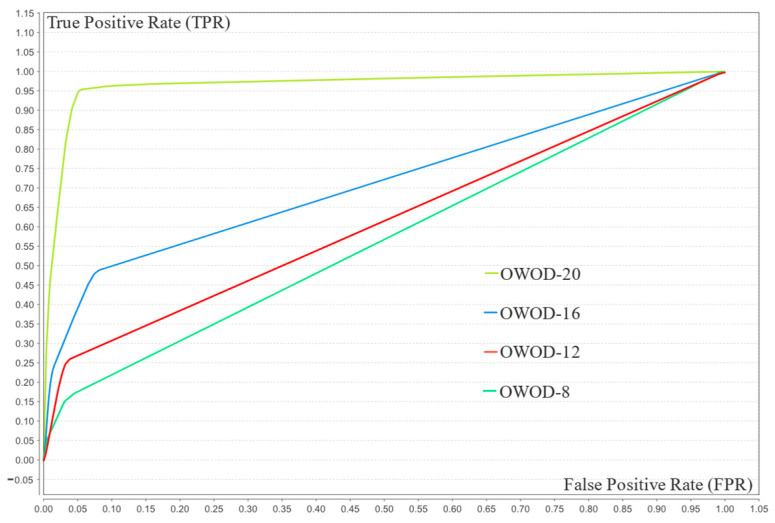
Receiver operating characteristic (ROC) chart of optimized weighted objective distance (OWOD) 8–20 features comparing the OWOD with 8 features (green), 12 features (red), 16 features (blue), and 20 features (lime).

As seen in Table 5, the model’s performance improved with an increasing number of features. At eight features, its performance was limited by low recall (15.68%) and F1-score (26.44%), indicating poor sensitivity. As the number increased to 12 and 16, recall and F1-score improved notably, reflecting enhanced balance. With 20 features, all metrics—accuracy (94.95%), precision (95.64%), recall (94.20%), F1-score (94.91%), and AUC (96.60%)—reached optimal levels. This demonstrates that higher feature dimensionality significantly boosts the model’s effectiveness, particularly in detecting minority class instances. The comparison of the ROC curves is illustrated in Figure 9.

The OWOD configuration with 20 features achieved the highest and most stable TPR across all FPR values, suggesting that 20 features represent the optimal balance between model complexity and performance.

### 4.2. OWOD Classification Results

Table 6 shows the results of the OWOD classification using 20 features. These are labeled as “dementia” for positive class groups and “heart failure” for negative groups.

These results demonstrate the ${OWOD}_{i}$ value and weight information for each person. The weight information indicates the degree to which a feature contributes to developing target conditions by representing the actual effects of each feature. Weight information with a value of 0 refers to an insignificant feature indicating control at the accepted level. Accordingly, the insignificant feature was eliminated to enhance classification performance. Weight information with a value of more than 0 refers to a significant feature affecting dementia development.

In addition, information gain computed from a reduction in entropy was employed to assign weights to each feature. The weighted features represent a degree of feature priority affecting classification. The summation of the OWOD of all features can be utilized to classify groups. Older adults with $OWOD\leq {OWOD}_{c}$ can be considered as belonging to the positive group (dementia), and those with $OWOD>{OWOD}_{c}$ to the negative group (heart failure). Thus, Person No. 1, with OWOD = 0.4051 and ${OWOD}_{c}=0.4324,$ was classified as belonging to the positive group.

#### Confusion Matrix Evaluation

The classification performance of the OWOD was also evaluated using a confusion matrix, as shown in Table 7. The classified class was derived from the proposed measurement, and the actual class was obtained from the doctor’s decision record. TP denotes true positive, representing older adults correctly classified as belonging to the OC group. TN denotes true negative, representing those correctly classified as belonging to the NC group. FP denotes a false positive, representing those incorrectly predicted as being in the OC group. FN denotes a false negative, representing those incorrectly predicted as being in the NC group.

The confusion matrix of the proposed OWOD investigating 8000 records reveals that 7596 people were correctly classified. This suggests that prioritizing features by applying different weights obtained from information gain to eliminate the features that do not influence the classification is feasible. In total, 404 individuals were incorrectly classified. Variability in a particular feature measurement may account for these inaccurate results. For instance, blood pressure was recorded manually; thus, the circumstances during measurement could affect exactness. Recent caffeine use or smoking can raise SBP readings above the baseline. These findings demonstrate the possibility of misclassifications and emphasize the difficulty in classifying cases with shared overlapping risk factors, especially when the input features lack adequate discriminatory power.

### 4.3. Model Validation Results

This study used five-fold cross-validation to verify the OWOD model. A comparison of the OWOD and ML classification models for the validation process is shown in Table 8.

The validation results demonstrate the superior performance of the OWOD compared with traditional and ensemble ML methods using 20 features. The OWOD achieved the highest overall performance across all key metrics, with an accuracy of 94.95% ± 0.96, a precision of 95.64% ± 0.95, a recall of 94.20% ± 1.11, an F1-score of 94.91% ± 0.48, and an AUC-ROC of 96.60% ± 0.013. GB, while relatively precise (91.34% ± 1.16), underperformed in recall and F1-score, indicating limitations in sensitivity. The DT, SVM, and NN models exhibited even lower recall and AUC-ROC values, suggesting a weaker ability to detect positive dementia cases accurately. These findings underscore the robustness and effectiveness of the OWOD, particularly its capacity to maintain high discrimination power, which is critical for reliable medical classification.

**Figure 10:**
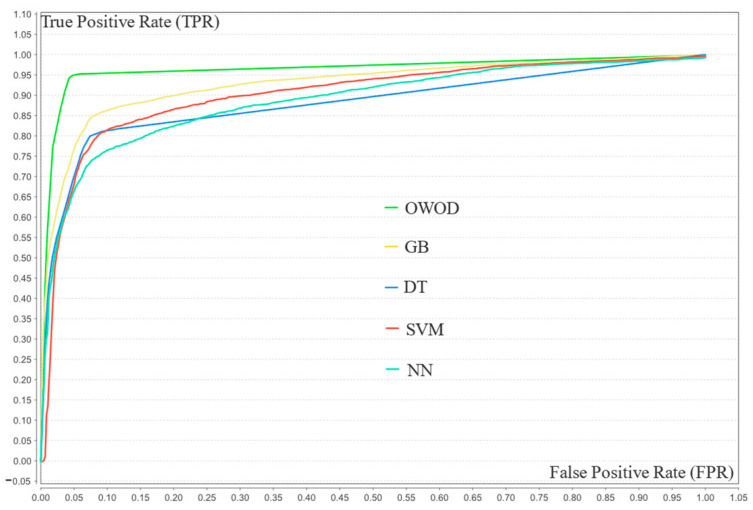
Receiver operating characteristic (ROC) curve comparison of the validation results of the optimized weighted objective distance (OWOD) and other machine learning models.

Figure 10 presents the ROC curves comparing the classification performance of five models—OWOD, GB, NN, DT, and SVM—using 20 features. The OWOD (green curve) outperformed all other methods with a consistently higher true positive rate (TPR) across nearly all false positive rates (FPRs), approaching a near-perfect classification boundary. The traditional ML models, particularly SVM and DT, demonstrated lower TPRs at equivalent FPRs, suggesting a reduced ability to distinguish between classes. This indicates that the OWOD offers a more effective solution for accurate classification in this context.

To ensure fair and consistent model comparisons, each method was implemented with carefully selected hyperparameters and evaluated using five-fold cross-validation. The hyperparameter details of both the ML and OWOD classifications are presented in Table 9. These configurations, outlined in Table 10, were selected based on grid search to ensure reliable and reproducible performance evaluation across models. 

### 4.4. Statistical Significance Comparison

**Figure 11:**
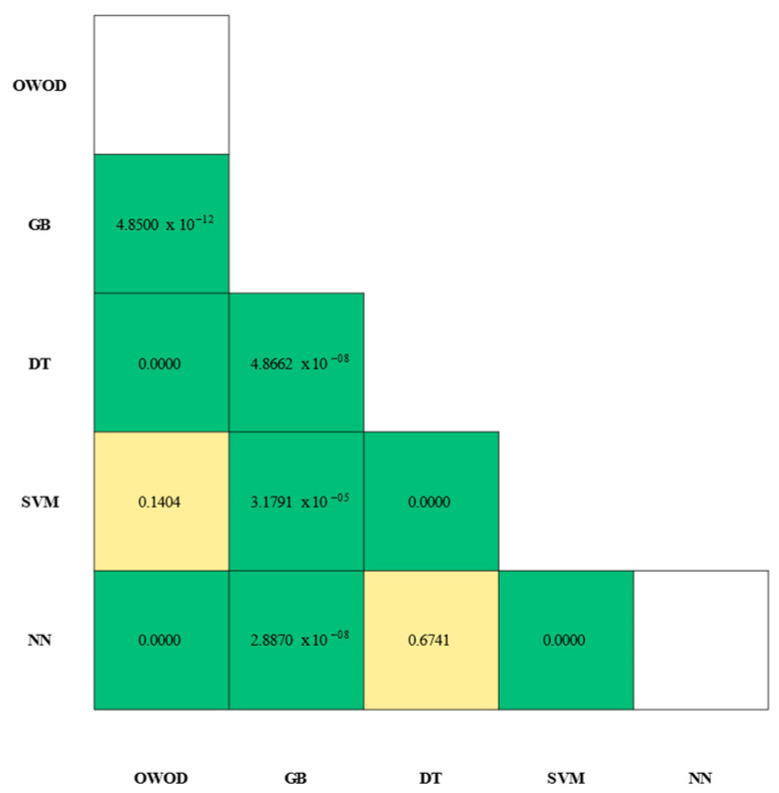
Statistical significance matrix from McNemar’s tests comparing model classifier pairs. Each cell represents the *p*-value of the comparison between two models. Green cells indicate statistically significant differences (*p* < 0.01), whereas yellow cells indicate non-significant differences (*p* ≥ 0.01).

Statistical validation of the model’s performance was carried out by pairing classifiers. A McNemar’s test was applied to the paired predictions of all models. The significance matrix is illustrated in Figure 11.

The results showed a statistically significant value for OWOD–GB ($p=4.85\times 10^{-12}$) in which the OWOD had the first-place performance (OWOD AUC = 96.60%; GB AUC = 92.90%), with GB’s performance showing significant differences against all models (p<0.01). In contrast, there were some overlapping efficiencies in the classification of OWOD–SVM (p= 0.1404) and DT–NN (p= 0.6741).

### 4.5. Model Comparison and Discussion

A performance comparison of the OWOD and other ML-based methods is presented in Table 10. These results were obtained from the evaluation using 20% unseen data.

**Table 10:** Comparison of the testing results of the optimized weighted objective distance (OWOD) and other machine learning models.

Classification Method	No. of Features	Accuracy %	Precision %	Recall %	F1-Score %	AUC-ROC %
OWOD	20	95.45	96.14	94.70	95.42	97.10
Gradient boosting (GB)	20	88.20	90.90	84.90	87.80	92.40
Decision tree (DT)	20	86.20	91.04	80.30	85.33	87.30
Support vector machine (SVM)	20	84.40	84.89	83.70	84.29	90.10
Neural network (NN)	20	83.75	88.14	78.00	82.76	88.80

The testing results confirm the superior classification performance of the OWOD compared with conventional ML algorithms. The OWOD achieved the highest scores across all evaluation metrics, with an accuracy of 95.45%, a precision of 96.14%, a recall of 94.70%, an F1-score of 95.42%, and an AUC-ROC of 97.10%. This reflects the ability of the OWOD to accurately identify positive and negative classes, maintaining a high balance between sensitivity and specificity. GB demonstrated reasonably strong precision (90.90%) but a poorer recall (84.90%) and F1-score (87.80%), indicating a greater likelihood of missing TP cases. Traditional models such as DT, SVM, and NN exhibited even lower recall and AUC-ROC values, indicating limited effectiveness in differentiating between classes. These findings demonstrate that the OWOD delivers high classification performance and offers enhanced generalization capability when applied to previously unseen data.

#### Evaluation of Model Performances

The results from both the validation and testing phases demonstrated the superior performance and robustness of the OWOD classification model over conventional ensemble and traditional ML models. During validation, the OWOD achieved the highest metrics across all performance indicators (accuracy 94.95% ± 0.96; precision 95.64% ± 0.95; recall 94.20% ± 1.11; F1-score 94.91% ± 0.48; AUC-ROC 96.60% ± 0.013). The OWOD maintained top-tier performance in the testing phase with an accuracy of 95.45%, a precision of 96.14%, a recall of 94.70%, an F1-score of 95.42%, and an AUC-ROC of 97.10%, demonstrating excellent generalization to unseen data.

While ensemble methods such as GB are known for improving accuracy by combining multiple weak learners, the OWOD surpassed GB in all metrics. This suggests that the OWOD’s class-wise feature interpretation and target-based thresholding mechanism enable a more refined and interpretable classification boundary. Despite leveraging ensemble principles, GB showed relatively lower recall (85.26% ± 1.38 in validation; 84.90% in testing), indicating potential underperformance in identifying TP cases—an important consideration in health-related applications where sensitivity is crucial.

Traditional classifiers such as DT, SVM, and NN exhibited further limitations, with lower recall, F1-score, and AUC-ROC values in both validation and testing. While computationally efficient, these models often struggle with complex decision boundaries and noisy/overlapping feature distributions. The OWOD incorporates a distribution-based entropy framework and utilizes optimal thresholds derived from class-wise histograms, allowing for the capture of fine-grained patterns that rigid or margin-based decision mechanisms in DT, SVM, and NN may overlook. In this context, the OWOD outperformed ensemble and traditional classifiers in predictive accuracy and robustness.

It can therefore be concluded that the proposed OWOD has a higher potential for classifying groups of individuals with dementia and heart failure compared with other ML classification methods. The assumption of this study is consequently verified, in that the proposed measurement can improve classification performance. In addition, the OWOD can handle complex attributes with multiple conditions during the attribute weighting process, which further enhances performance.

### 4.6. Suggestions and Future Study

This study demonstrated the effectiveness of the proposed OWOD classification method in classifying individuals with dementia and heart failure using blood biomarker features. The model achieved strong performance across all evaluation metrics, underscoring its potential as a practical, noninvasive, and widely accessible decision support tool. To support clinical adoption, future research should focus on validating the model across diverse populations and healthcare settings, as well as exploring additional blood-based biomarkers to further enhance performance. While these findings are promising, the exclusion of individuals diagnosed with both dementia and heart failure in order to maintain clear class boundaries is a limitation and may reduce applicability in real-world settings. Incorporating more granular clinical information and multi-label classification approaches would allow for more sophisticated modeling and improved classification performance among complex patient populations.

The OWOD offers clear interpretability, as its decisions are based on the distance from target feature values, allowing clinicians to understand the contribution of each factor. Additionally, the OWOD can offer personalized recommendations according to each individual’s significant features after classification as efficiently as healthcare providers. These features could be used to guide lifestyle changes on a broad scale. As a result, older adults could better monitor their health status, which may lead to dementia/heart failure prevention. Potential barriers to real-world implementation include validation across diverse populations, seamless integration with existing EHR systems, and acceptance by clinicians and patients.

## 5. Conclusions

This study proposes the OWOD as a novel measurement to distinguish groups of patients with dementia and heart failure. The OWOD is a modification of the original WOD using entropy-based weighting features and an objective function from class-wise histogram differences, promoting a data-driven approach. The weighted features indicate a degree of feature priority affecting classification and eliminate features that do not influence classification. This study evaluated the proposed OWOD using 20 features and 10,000 EHRs and compared it with other ML classification models, including GB, DT, SVM, and NN. The OWOD outperformed the other ML models during validation and testing processes for overall performance comparison in terms of accuracy, precision, recall, F1-scores, and AUC-ROC. Future studies should include more multi-site datasets and refine features to ensure generalization and practical applicability.

## Figures and Tables

**Table 1:** Summary of dementia and heart failure risk factors identified in previous studies.

Dementia	Heart Failure
Body weight [32]	Being overweight/obese [46]
Blood cholesterol [33]	Hypercholesterolemia [46]
Hypertension [29,30,31]	Hypertension [12,46]
Serum lipids [34]	Lipid biomarkers [47]
Diabetes [1]	Diabetes mellitus [46]
Sex [42]	Sex [12,46,47]
ApoE4 gene [16]	Age [12,46,47]
Air pollution (NO_2_, PM2.5) [1]	
Sleep disturbances [40]	

**Table 2:** Description of the features used in the current study.

Features	Acronym	Data Range	Group
Body weight (kg)	W	40.1–116.3	R
Height (cm)	H	150.1–185.0	P
Body mass index (kg/m^2^)	BMI	11.76–39.57	R
Systolic blood pressure (mmHg)	SBP	76–199	R
Diastolic blood pressure (mmHg)	DBP	61–126	R
Fasting blood sugar (mg/dL)	FBS	62–495	R
Triglycerides (mg/dL)	TGS	51–199	R
Total cholesterol (mg/dL)	TC	101–429	R
High-density lipoprotein cholesterol (mg/dL)	HDL	31–93	R
Low-density lipoprotein cholesterol (mg/dL)	LDL	51–196	R
Hemoglobin (g/dL)	HB	10.10–20.10	P
White blood cell (count/μL)	WBC	3100–19,900	P
Polymorphonuclear neutrophils (percentage)	NEUT	30.20–89.90	P
Thrombocytes (count/μL)	PLAT	101,000–585,000	P
Lymphocyte cells (percentage)	LYMP	10.10–59.00	P
Creatinine (mg/dL)	CREA	0.35–2.99	P
Blood urea nitrogen (mg/dL)	BUN	4–49	P
Thyroid stimulating hormone (mIU/L)	TSH	0.01–5.97	P
Potassium (mEq/L)	K	1.40–7.80	P
Sodium (mEq/L)	NA	109–167	P
Carbon dioxide (mEq/L)	CO_2_	11–45	P

**Table 3:** Sample target and acceptable values.

Feature (i)	Target Level ($T_{mi}$)	Acceptable Level ($A_{i}$)
W	55 kg	90 kg
SBP	150 mg/dL	190 mg/dL
DBP	85 mg/dL	110 mg/dL
FBS	130 mg/dL	280 mg/dL
TGS	140 mg/dL	160 mg/dL
TC	200 mg/dL	260 mg/dL
HDL	55 mg/dL	70 mg/dL
LDL	135 mg/dL	165 mg/dL

**Table 4:** Examples of the dataset.

No.	W	SBP	DBP	FBS	TGS	TC	HDL	LDL
1	65	130	75	120	115	175	35	105
2	70	140	80	105	95	165	50	120
3	55	155	90	110	110	195	60	140
4	65	135	75	100	100	185	40	120
5	50	150	75	115	155	205	50	135
6	60	135	85	115	100	185	60	145
7	92	155	85	120	125	220	50	140
8	77	160	80	115	100	210	50	135
9	47	121	82	130	85	215	55	130
10	45	162	77	115	120	205	60	125
…	…	…	…	…	…	…	…	…
8000	65	135	75	100	100	185	40	120

**Table 5:** Comparison of optimized weighted objective distance classifications by feature selection number.

No. of Features	Accuracy %	Precision %	Recall %	F1-Score %	AUC-ROC %
8	56.38 ± 1.94	85.05 ± 3.04	15.68 ± 5.13	26.44 ± 0.97	56.40 ± 0.02
12	61.26 ± 1.52	89.26 ± 1.84	25.62 ± 3.48	39.81 ± 0.76	61.40 ± 0.016
16	70.43 ± 0.28	86.61 ± 0.86	48.34 ± 0.86	62.05 ± 0.14	71.30 ± 0.003
20	94.95 ± 0.96	95.64 ± 0.95	94.20 ± 1.11	94.91 ± 0.48	96.60 ± 0.013

**Table 6:** Optimized weighted objective distance (OWOD) classification results.

No.	Results											Matching Result
	Weight								OWOD	Classification	Actual	
	SBP	DBP	W	FBS	TGS	TC	HDL	LDL	Result	Result	Result	Correct /Incorrect
1	0.0003	0.0003	0.1404	0.0531	0.2095	0.1010	0.2582	0.2371	0.4051	Dementia	Dementia	Correct
2	0.1034	0.0003	0.1117	0.1216	0.1484	0.1484	0.1995	0.1667	0.4258	Dementia	Dementia	Correct
3	0.2727	0.0004	0.0004	0.0611	0.2484	0.0004	0.2409	0.1757	0.4057	Dementia	Dementia	Correct
4	0.1920	0.2168	0.0003	0.0467	0.2251	0.0003	0.1843	0.1344	0.5858	Dementia	Heart failure	Incorrect
5	0.2555	0.0005	0.2705	0.1849	0.0005	0.2872	0.0005	0.0005	0.3583	Dementia	Dementia	Correct
6	0.1356	0.0960	0.1301	0.0815	0.1149	0.1476	0.1475	0.1468	0.4822	Heart failure	Heart failure	Correct
7	0.1364	0.1225	0.0772	0.1281	0.1417	0.1378	0.1152	0.1411	0.4411	Heart failure	Heart failure	Correct
8	0.1465	0.1284	0.1137	0.0654	0.1207	0.1366	0.1444	0.1444	0.8029	Heart failure	Heart failure	Correct
9	0.1231	0.1257	0.1283	0.0862	0.1181	0.1337	0.1435	0.1414	0.6858	Heart failure	Heart failure	Correct
10	0.1456	0.1446	0.1158	0.0511	0.1192	0.1229	0.1515	0.1493	0.5363	Heart failure	Heart failure	Correct
…	…	…	…	…	…	…	…	…	…	…	…	…
8000	0.1265	0.1292	0.1144	0.0973	0.1374	0.1081	0.1497	0.1374	0.4680	Heart failure	Dementia	Incorrect

**Table 7:** Confusion matrix of the proposed optimized weighted objective distance.

	Dementia	Heart Failure	Class Precision
Predict—Dementia	3768 (TP)	172 (FP)	95.63%
Predict—Heart failure	232 (FN)	3828 (TN)	94.29%
Class recall	94.20%	95.70%	

**Table 8:** Comparison of the validation results of the optimized weighted objective distance (OWOD) and other machine learning models.

Classification Method	No. of Features	Accuracy %	Precision %	Recall %	F1-Score %	AUC-ROC %
OWOD	20	94.95 ± 0.96	95.64 ± 0.95	94.20 ± 1.11	94.91 ± 0.48	96.60 ± 0.013
Gradient boosting (GB)	20	88.58 ± 0.77	91.34 ± 1.16	85.26 ± 1.38	88.19 ± 0.39	92.90 ± 0.006
Decision tree (DT)	20	86.75 ± 0.72	91.83 ± 1.51	80.72 ± 1.59	85.90 ± 0.36	88.10 ± 0.005
Support vector machine (SVM)	20	84.96 ± 1.03	85.81 ± 1.03	83.78 ± 1.91	84.78 ± 0.52	90.70 ± 0.007
Neural network (NN)	20	83.34 ± 0.87	88.19 ± 2.41	77.08 ± 1.59	82.23 ± 0.44	89.30 ± 0.006

**Table 9:** Summary of the hyperparameters of the machine learning classifications.

Method	Hyperparameters
Gradient boosting	Number of trees = 50; maximal depth = 5; min rows = 10.0; number of bins = 20; learning rate = 0.01; sample rate = 1.0; cross-validation folds = 5
Decision tree	Criterion = gain ratio; maximal depth = 10; confidence = 0.1; minimal gain = 0.01; minimal leaf size = 2; cross-validation folds = 5
Support vector machine	Kernel type = dot; kernel cache = 200; C = 0.0; convergence epsilon = 0.001; max iterations = 100,000; cross-validation folds = 5
Neural network	Hidden layer = 2; training cycle = 200; learning rate = 0.01; momentum = 0.9; cross-validation folds = 5
OWOD	Cross-validation folds = 5

## Data Availability

Access to the dataset is currently restricted, as the study is still ongoing.
